# Soluble Poly(amide-imide)s from Diamide–Diamine Monomer with Trifluoromethyl Groups

**DOI:** 10.3390/polym14030624

**Published:** 2022-02-06

**Authors:** Taejoon Byun, Seong Jong Kim, Sang Youl Kim

**Affiliations:** Department of Chemistry, Korea Advanced Institute of Science and Technology (KAIST), Daejeon 34141, Korea; ruminaire@naver.com (T.B.); krisj@kaist.ac.kr (S.J.K.)

**Keywords:** high-temperature polymer, poly(amide-imide)s, trifluoromethyl group

## Abstract

A series of soluble aromatic poly(amide-imide)s (PAIs) was prepared from a new diamide–diamine monomer having biphenyl units with two CF_3_ groups. The diamide–diamine monomer was polymerized with 2,2′-bis(trifluoromethyl)benzidine and pyromelltic dianhydride through an imidization reaction to prepare PAIs with a controlled imide/amide bond ratio in the main chains. While the PAIs with the highest imide bond content showed a limited solubility, other PAIs were soluble in polar organic solvents and can be solution-cast into flexible freestanding films. All PAIs exhibited high thermal stability with 5% weight loss temperature (*T*_d5_) from 464 to 497 °C in air, and no appearance of glass transition up to 400 °C. Notably, the linear coefficient of thermal expansion (CTE) value of the PAI films was linearly decreased with the imide bond content and varied from 44.8 to 7.8 ppm/°C.

## 1. Introduction

Current rapid technological developments in display and microelectronic devices demand processable high-performance materials which have thermo-dimensional stability. Polyimides are one of processable high-performance polymers that can meet these requirements, but soluble poly(amic acid)s are often used as a precursor for processing and then transformed to polyimides by thermal curing. [1]. Though polyimides possess many attractive features, most of the wholly aromatic polyimides with rigid structures for thermo-dimensional stability generally suffer poor solubility in organic solvents, which often frustrates their diverse application [2]. To overcome this problem, various structural modifications of polyimides have been attempted over the last few decades [3,4,5,6,7,8].

Aromatic poly(amide-imide)s (PAIs) are one of such variants that developed as alternative materials for polyimides with a promising balance of thermal stability and processability [9]. By converting some of the imide groups in the polymer chains to amides, PAIs achieve improved solubility and processability without compromising their excellent thermal and mechanical properties, thanks to the hydrogen bonding of amide groups [10,11,12]. Among many synthetic approaches on structurally modified PAIs, the introduction of biphenylene units with trifluoromethyl (-CF_3_) groups is found to be effective in improving solubility and optical properties of PAIs without deterioration of thermal properties [13,14,15].

In the synthetic perspective, most aromatic PAIs are prepared via two synthetic routes: one is direct polycondensation between diamine and trimellitic anhydride or trimellitic anhydride chloride, and the other is the imidization/amidation of amide-/imide-containing monomers. While the former route is usually employed in commercial production of PAIs [16,17], the majority of laboratory studies utilize the latter route because polymerization can be readily conducted through conventional synthetic techniques used for polyimides or polyamides [18,19]. Particularly, most structural modifications of PAIs have been achieved by the development and subsequent polymerization of novel diimide-dicarboxylic acid monomers which can be easily prepared from diamine and commercially available trimellitic anhydride via simple refluxing in glacial acetic acid [20,21]. In contrast, imide- or amide-containing diamines have not been developed extensively, presumably because of relative synthetic inconvenience [22].

The use of amide-containing diamine as a monomer for PAIs has a certain advantage over diimide-dicarboxylic acid because the polymer can be obtained by conventional imidization process, which is cost-effective and also suitable for coating applications compared to the amidation process usually employed for diimide-dicarboxylic acid. Moreover, the ratio between the imide bond and amide bond within a PAI structure can be deliberately adjusted while keeping the imide bond as a major component, enabling the precise modulation of polymer properties with minimum loss of the characteristics of polyimides [23].

In this study, we designed a new diamide–diamine monomer containing two biphenylene units with CF_3_ groups and synthesized the PAIs through imidization with pyromellitic dianhydride. We also prepared a series of copolymers with different imide/amide bond compositions through copolymerization of the new monomer with 2,2′-bis(trifluoromethyl)benzidine and investigated the effect of bond ratio on solubility and thermal properties of PAIs. The incorporation of amide bonds together with multiple CF_3_ groups would impart good organosolubility and thermal properties to the corresponding PAIs.

## 2. Materials and Methods

### 2.1. Materials

4′-Nitro-2′,6′-bis(trifluoromethyl)-[1,1′-biphenyl]-4-amine (**1**) was synthesized as previously reported in our paper [14]. Isophthaloyl chloride was purchased from TCI (Tokyo Chemical Industry Co. Ltd., Tokyo, Japan) and used as received. 2,2′-Bis(trifluoromethyl)benzidine (TFMB) and pyromellitic dianhydride (PMDA) were also purchased from TCI and purified by vacuum sublimation at 120 °C and 165 °C, respectively. Tetrahydrofuran (THF) was deoxygenated and dried by purging with nitrogen gas followed by passage through an activated alumina column. *m*-Cresol and *N*-methyl-2-pyrrolidone (NMP) were purchased from Daejung (Siheung, Korea) and purified by distillation under reduced pressure over phosphorus pentoxide. All other commercially available reagent-grade chemicals were used without further purification.

### 2.2. Measurements

The NMR spectrum of the compounds prepared in this study was taken on a Bruker Fourier Transform Advance 400 spectrometer (Bruker, Billerica, MA, USA). The NMR chemical shift was reported in parts per million (ppm) using tetramethylsilane as an internal standard. Splitting patterns of the peaks were designated as a s (singlet), d (doublet), dd (doublets of doublet), t (triplet), q (quartet), or m (multiplet). The Fourier transform infrared (FT-IR) spectra of the compounds were obtained with a Thermo Fisher Scientific Nicolet iS50 FTIR spectrophotometer (Thermo Fisher, Waltham, MA, USA). The inherent viscosity of the polymers was measured using an Ubbelohde viscometer (Witeg, Wertheim, Germany). Size exclusion chromatography (SEC) trace was obtained using an Agilent 1260 Infinity system (Agilent Technologies, Santa Clara, CA, USA) equipped with an Optilab T-rEX refractive index detector and three PLgel 10 μm Mixed-B columns using DMF containing 0.05 M LiBr as an eluent, at 40 °C. The molecular weight of the synthesized polymer was calculated relative to the linear polystyrene standards. Thermogravimetric analysis (TGA) was performed in a TA Instruments TGA Q50 (TA Instruments, New Castle, DE, USA) in the temperature range of 40 °C to 800 °C at a heating rate of 10 °C/min under nitrogen and air atmosphere, respectively. The used sample mass for analysis was 5~10 mg. Differential scanning calorimetry (DSC) were performed on a DSC Q20 instrument (TA Instruments, New Castle, DE, USA) in the temperature range of 0 °C to 400 °C at a scanning rate of 10 °C/min under nitrogen atmosphere. The used sample mass for analysis was 5~10 mg. Glass transition temperature (*T*_g_) was taken from the second heating scan. The linear coefficients of thermal expansion (in-plane CTE) of polymer films were measured by thermal mechanical analysis (TMA) using a TA TMA-Q400 thermomechanical analyzer (TA Instruments, New Castle, DE, USA). The measurements were carried out three times in a heating range up to 300 °C at a heating rate of 5 °C/min, and CTE value was evaluated as an average rate of change in the temperature range of 50 °C to 250 °C in the second and third heating runs. Mechanical properties of the PAI films were measured by universal testing machine (UTM) using a 68SC-1 INSTRON (INSTRON, Norwoodm, MA, USA). UV-visible spectrum was taken with a Shimadzu UV-2600 spectrometer (Shimadzu, Kyoto, Japan) in transmittance mode. The refractive index for transverse electric (TE, *n*_TE_) and transverse magnetic (TM, *n*_TM_) modes of the polymer films were obtained by using a Sairon SPA-4000 prism coupler (Sairon Technology, Gwangju, Korea) with a gadolinium gallium garnet (GGG) prism at 633 nm and 1310 nm at RT. The birefringence values (Δ*n*) were calculated as the difference between *n*_TE_ and *n*_TM_. Wide-angle X-ray diffraction (WAXD) measurements were performed at room temperature on Rikagu SMARTLAB X-ray diffractometer (Rigaku, Tokyo, Japan) with a Cu Kα1 incident beam. Matrix-assisted laser absorption ionization time-of-flight (MALDI-TOF) mass spectroscopy study was performed on a Bruker autoflex maX (Bruker, Billerica, MA, USA) using reflector mode for operation. Ionization was performed with a 355 nm pulsed Nd YAG laser, and samples were prepared with dithranol as a matrix and sodium trifluoroacetate as an ion source.

### 2.3. Monomer Synthesis

*N*^1^,*N*^3^-Bis(4′-nitro-2′,6′-bis(trifluoromethyl)-[1,1′-biphenyl]-4-yl)isophthalamide (**2**). A 100 mL two-neck round-bottom flask was charged with **1** (2.5216 g, 7.20 mmol), pyridine (0.6 mL, 7.42 mmol), and 20 mL of THF. The flask was sealed with a rubber septum and subsequently degassed and backfilled with nitrogen. The mixture was stirred at room temperature until the solution became homogeneous. Then, a solution of isophthaloyl chloride (0.7309 g, 3.60 mmols) in THF (10 mL) was added dropwise to the stirring mixture by syringe pump. After the addition, the reaction mixture was additionally stirred for 6 h at room temperature. Upon completion, the reaction was quenched with 1 N HCl and the mixture was subsequently washed with brine. The organic layer was dried over MgSO_4_ and solvent was removed in a rotary evaporator. The resulting crude mixture was recrystallized in toluene to give beige-colored solid product **2** (2.8138 g, 3.39 mmols, 94.1% yield). m.p. 253–254 °C. FT-IR (powder, cm^−1^): 3226 (amide N-H); 1649 (amide C=O); 1537, 1331 (NO_2_); 1176, 1142, 1126 (C-F in CF_3_). ^1^H NMR (DMSO-*d*_6_, 400 MHz, ppm): 10.67 (s, amide, 2H), 8.81 (s, 4H), 8.59 (t, *J* = 1.6 Hz, 1H), 8.19 (dd, *J* = 7.8, 1.8 Hz, 2H), 7.93 (d, *J* = 8.8 Hz, 4H), 7.75 (t, *J* = 7.8 Hz, 1H), 7.34 (d, *J* = 8.4 Hz, 4H). ^13^C NMR (DMSO-*d_6_*, 100 MHz, ppm): 165.86, 147.43, 146.23, 140.38, 135.58, 132.17 (q, *J* = 30.5 Hz), 131.35, 130.27, 129.24, 127.61, 127.41, 125.37 (q, *J* = 5.4 Hz), 122.64 (q, *J* = 273.5 Hz), 119.25.

*N*^1^,*N*^3^-Bis(4′-amino-2′,6′-bis(trifluoromethyl)-[1,1′-biphenyl]-4-yl)isophthalamide (**3**). A 250 mL Schlenk flask was charged with **2** (2.50 g, 3.01 mmol), 10% Pd/C (1 g), and 70 mL of the mixture of ethanol/ethyl acetate (*v*/*v* = 1:1). The reaction mixture was evacuated and flushed with hydrogen. Then, the mixture was stirred at room temperature for 48 h under hydrogen atmosphere. The catalyst was removed using celite, and the solvent was evaporated using a rotary evaporator. To obtain a pale-yellow solid product, the crude product was passed down a silica column using ethyl acetate/hexane (*v*/*v* = 1:1) as an eluent and then recrystallized in anisole. The recrystallized product was re-dissolved in acetone and precipitated in *n*-hexane to remove residual anisole. The precipitate was dried in vacuo at 80 °C for 18 h to obtain **3** as off-white powder (2.00 g, 2.60 mmol, 86.2% yield). m.p. 352–353 °C. FT-IR (powder, cm^−1^): 3504, 3435, 3409, 3345 (NH_2_ stretching); 3248 (amide N-H); 1660 (amide C=O); 1631 (NH_2_ bending); 1167, 1113 (C-F in CF_3_). ^1^H NMR (DMSO-*d*_6_, 400 MHz, ppm): 10.50 (s, amide, 2H), 8.55 (t, *J* = 1.6 Hz, 1H), 8.16 (dd, *J* = 7.8, 1.7 Hz, 2H), 7.77 (d, *J* = 8.8 Hz, 4H), 7.71 (t, *J* = 7.8 Hz, 1H), 7.22 (s, 4H), 7.18 (d, *J* = 8.4 Hz, 4H), 6.09 (s, NH_2_, 4H). ^13^C NMR (DMSO-*d_6_*, 100 MHz, ppm): 165.21, 148.77, 138.59, 135.26, 131.15, 130.71, 130.62 (q, *J* = 28.0 Hz), 139.81, 128.69, 127.03, 124.33, 123.56 (q, *J* = 272.9 Hz), 118.69, 113.18 (q, *J* = 5.4 Hz).

### 2.4. Polymerization

**PAI-1.** A 25 mL three-necked round-bottom flask equipped with a mechanical stirrer, Dean-Stark trap and nitrogen inlet was charged with **3** (0.5009 g, 0.650 mmol) and *m*-cresol (4 mL). With mild nitrogen flowing, the mixture was gently stirred and heated until the diamine monomer was fully dissolved. Then, it was cooled down to room temperature and pyromellitic dianhydride (0.1420 g, 0.651 mmol) was added. After 30 min of stirring, isoquinoline (about 5 drops) was added, and the mixture was agitated for another 16 h at room temperature. After diluting the solution with 4 mL of m-cresol to 8 wt% concentration, the temperature was increased to 190°C and the reaction mixture was stirred for 12 h at this temperature. During this period, the water generated during the imidization process was extracted through distillation as a chlorobenzene/water azeotrope. After 2 h of polymerization, the solution started to become turbid and a small amount of *m*-cresol (total addition volume = 6 mL) was added periodically to prevent precipitation. At the end of the reaction, the heterogeneous polymer solution was cooled down to 100 °C and the hot solution was poured slowly into an excess amount of vigorously stirred methanol. The polymer was precipitated as a yellowish-brown solid and collected by filtration. The obtained polymer was washed thoroughly with methanol and dried overnight in vacuoat 80 °C (0.6107 g, 98.4% yield, η_inh_ = 0.86 dL/g). FT-IR (film, cm^−1^): 3363 (amide N-H stretching); 1782, 1728 (imide C=O); 1670 (amide C=O); 1520 (amide N-H bending); 1471 (aromatic C=C); 1363 (imide C-N stretching); 1174, 1128, 1093 (C-F in CF_3_); 823 (C-H bending of PMDA); 721 (imide ring deformation). ^1^H NMR (DMF-*d*_7_, 400 MHz, ppm): 10.75 (s, amide, 2H), 8.74 (s, 1H), 8.66 (s, 2H), 8.51 (s, 2H), 8.30 (d, *J* = 7.2 Hz, 2H), 8.15 (d, *J* = 8.2 Hz, 2H), 7.78 (t, *J* = 7.8 Hz, 1H), 7.53 (d, J = 8.5 Hz, 2H).

**PAI-2.** The same protocol used for **PAI-1** was repeated with the 1:1 molar mixture of **3** (0.3223 g, 0.419 mmol) and TFMB (0.1343 g, 0.419 mmol), pyromellitic dianhydride (0.1829 g, 0.838 mmol) and *m*-cresol (4 mL). Before heating the reaction mixture to 190 °C, the solution was diluted with 4 mL of *m*-cresol. In this case, the reaction mixture showed a good solubility and homogeneity during the polymerization process, and thus no additional amount of *m*-cresol was added (0.6077 g, 99.7% yield, η_inh_ = 0.94 dL/g). FT-IR (film, cm^−1^): 3367 (amide N-H stretching); 1782, 1724 (imide C=O); 1674 (amide C=O); 1522 (amide N-H bending); 1490, 1473, 1427 (aromatic C=C); 1363 (imide C-N stretching); 1174–1057 (C-F in CF_3_); 823 (C-H bending in PMDA); 721 (imide ring deformation). ^1^H NMR (DMF-*d*_7_, 400 MHz, ppm): 10.75 (s, amide, 2H), 8.75 (s, 1H), 8.66 (s), 8.62 (s), 8.58 (s), 8.53 (s), 8.51 (s), 8.30–8.26 (m, 4H), 8.16–8.10 (m, 6H), 7.89–7.87 (m, 2H), 7.79–7.76 (m, 2H), 7.53 (d, *J* = 8.2 Hz, 2H).

**PAI-3.** The same protocol used for **PAI-1** was repeated with the 1:3 molar mixture of **3** (0.1894 g, 0.246 mmol) and TFMB (0.2361 g, 0.737 mmol), pyromellitic dianhydride (0.2145 g, 0.983 mmol) and NMP (4 mL). In this case, the solvent was replaced with NMP instead of *m*-cresol because the limited solubility of resulting polymers was expected. Before heating the reaction mixture to 190 °C, the solution was diluted with 4 mL of NMP. After 4 h of polymerization, the solution started to become gelated and precipitation occurred eventually. The heterogeneous mixture was further stirred for 8 h with the occasional addition of NMP (total addition volume = 8.5 mL). The solution was put into extra methanol when it had cooled to room temperature. Solid polymer power was collected by filtration, washed with methanol, and dried overnight in vacuo at 100 °C (0.6176 g, 100% yield). FT-IR (film, cm^−1^): 3373 (amide N-H stretching); 1782, 1720 (imide C=O); 1684 (amide C=O); 1522 (amide N-H bending); 1491, 1475, 1425 (aromatic C=C); 1362 (imide C-N stretching); 1173–1057 (C-F in CF_3_); 823 (C-H bending in PMDA); 723 (imide ring deformation). ^1^H NMR (DMF-*d*_7_, 400 MHz, ppm): 10.75 (s, amide, 2H), 8.75 (s, 1H), 8.66 (s), 8.62 (s), 8.58 (s), 8.52 (s, 4H), 8.30–8.26 (m, 2H), 8.16–8.09 (m, 8H), 7.89–7.86 (broad, 6H), 7.79 (broad, 1H), 7.53 (d, *J* = 6.9 Hz, 2H).

### 2.5. Preparation of Poly(amide-imide) Films

**PAI-1, PAI-2.** A total of 0.1 g of the polymer was dissolved in 5 mL of DMAc at room temperature; then, the homogeneous solution was passed through 5.0 μm PTFE syringe filter and drop casted on 5 × 5 cm glass plate. The solvent was slowly evaporated in a vacuum oven at 40 °C for 2 h; then, the obtained polymer film was thermally cured at 200 °C for 1 h and 300 °C for 2 h.

**PAI-3.** A total of 0.1 g of the polymers was dissolved in 10 mL of NMP with gentle heating, then the solutions were passed through 5.0 μm PTFE syringe filter and drop casted on PTFE Petri dish (*φ* = 5 cm). The solvent was slowly evaporated in vacuum oven at 60 °C for 2 h, and then the obtained polymer film was thermally cured at 200 °C for 1 h and 300 °C for 2 h.

## 3. Results and Discussion

### 3.1. Monomer Synthesis

**Scheme 1:**
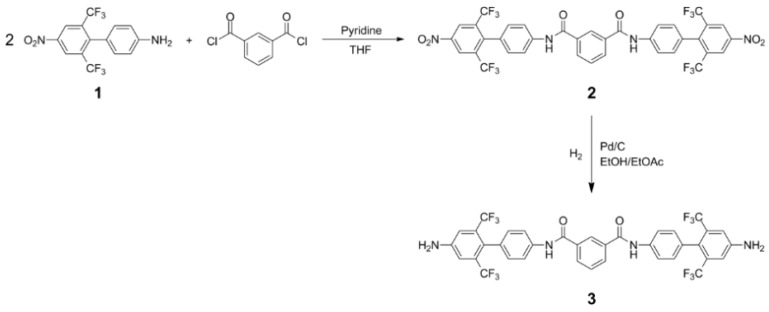
Synthesis of the diamide–diamine monomer.

The diamide–diamine monomer **3** was prepared from our previously developed nitro-amine compound **1** as depicted in Scheme 1. In the first step, isophthaloyl chloride was reacted with two equivalents of monofunctional nitro-amine compound **1** in the presence of pyridine as an acid acceptor to obtain the diamide–dinitro compound **2**. Subsequently, the dinitro compound was transformed to the corresponding diamine monomer **3** by hydrogenation in the presence of Pd/C catalyst.

**Figure 1:**
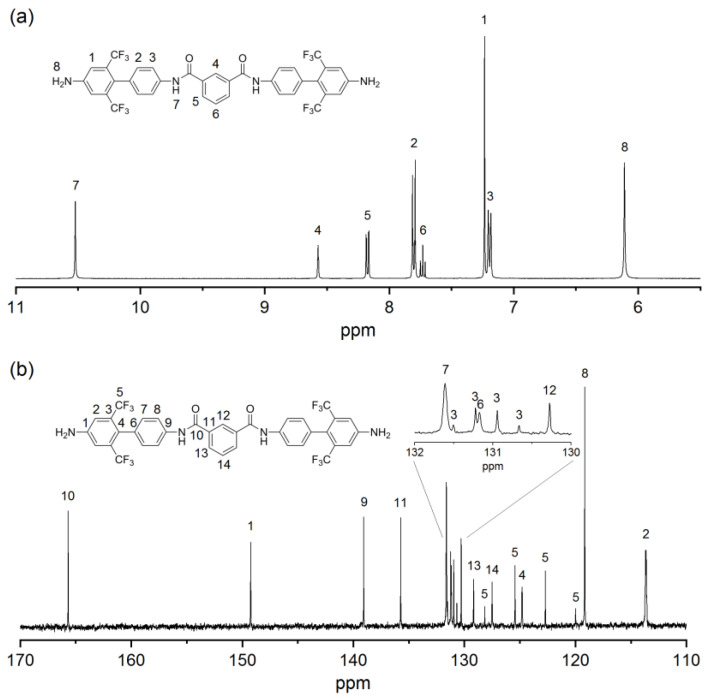
(**a**) ^1^H and (**b**) ^13^C NMR spectra of diamide-diamine **3** (DMSO-*d*_6_, 25 °C).

The structures of dinitro compound **2** and diamine monomer **3** were verified by FTIR, MALDI–TOF (Figure S11), ^1^H and ^13^C NMR. Figure S1 shows the FT-IR spectra of **1**, **2** and **3**. Sharp absorption peaks of amine group (3498, 3400 cm^−1^) in **1** disappeared after the amidation, and **2** gave characteristic absorption bands corresponding to amide and nitro groups at 3226 cm^−1^ (amide N-H stretching), 1649 cm^−1^ (amide C=O stretching), 1537 cm^−1^ and 1331 cm^−1^ (NO_2_ asymmetric and symmetric stretching). After reduction, the characteristic absorptions of the nitro groups disappeared, and the amino group of **3** exhibited several N-H stretching bands in the regions of 3345–3504 cm^−1^ together with N-H bending band at 1631 cm^−1^. The ^1^H and ^13^C NMR spectra of **2** and **3** are shown in Figure S2 and Figure 1, respectively. All the proton and carbon peaks were well assigned to their predicted structure. The proton resonance at 10.50 and 6.04 ppm in **3** confirms the existence of amide and amine groups in the diamide–diamine monomer.

### 3.2. Polymerization

**Scheme 2:**
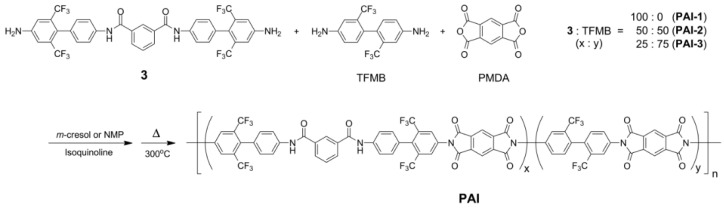
Synthesis of new poly(amide-imide)s.

A series of poly(amide-imide)s were prepared from **3**, TFMB and pyromellitic dianhydride (PMDA) through the one-pot method of solution imidization, as shown in Scheme 2. The polymerizations of diamines with a stoichiometric amount of PMDA were performed in *m*-cresol or NMP with catalytic amount of isoquinoline with solid content around 16 wt%. The ring opening and addition reactions at RT for 16 h yield poly(amic acid)s solutions. Following dilution of the solution to 8% (weight), cyclodehydration was performed at 190 °C for 12 h to remove water through azeotropic distillation with chlorobenzene. While **PAI-2** maintained homogeneity during the polymerization, **PAI-1** and **PAI-3** were precipitated in the middle of polymerization presumably because of the weak affinity of **3** with *m*-cresol and high chain rigidity of **PAI-3**. When the polymerization was complete, the solid products were obtained by precipitating the polymer solution into excess methanol. Subsequent ^1^H NMR analysis revealed that all the obtained polymers possessed a small portion of amic acid structure, indicating the imidization was not completed. This might be attributed to the premature precipitation as well as weakened nucleophilicity of **3** due to the presence of strong electron withdrawing in two CF_3_ groups. Therefore, additional thermal curing was conducted at 300 °C in order to achieve full imidization. The inherent viscosity of thermally cured **PAI-1** and **PAI-2** were 0.86 dL/g and 0.94 dL/g, respectively. The inherent viscosity of **PAI-3** could not be measured because it was only soluble in hot organic solvent and quickly became gelated upon cooling. The average molecular weights of polymers, **PAI-1** and **PAI-2**, were measured by GPC by using DMF/LiBr as an eluent. The number average molecular weights of **PAI-1** and **PAI-2** were 37.9 kDa and 35.0 kDa, respectively, and the weight average molecular weights of **PAI-1** and **PAI-2** were 88.1 kDa and 84.9 kDa, respectively (Figures S3 and S4).

**Figure 2:**
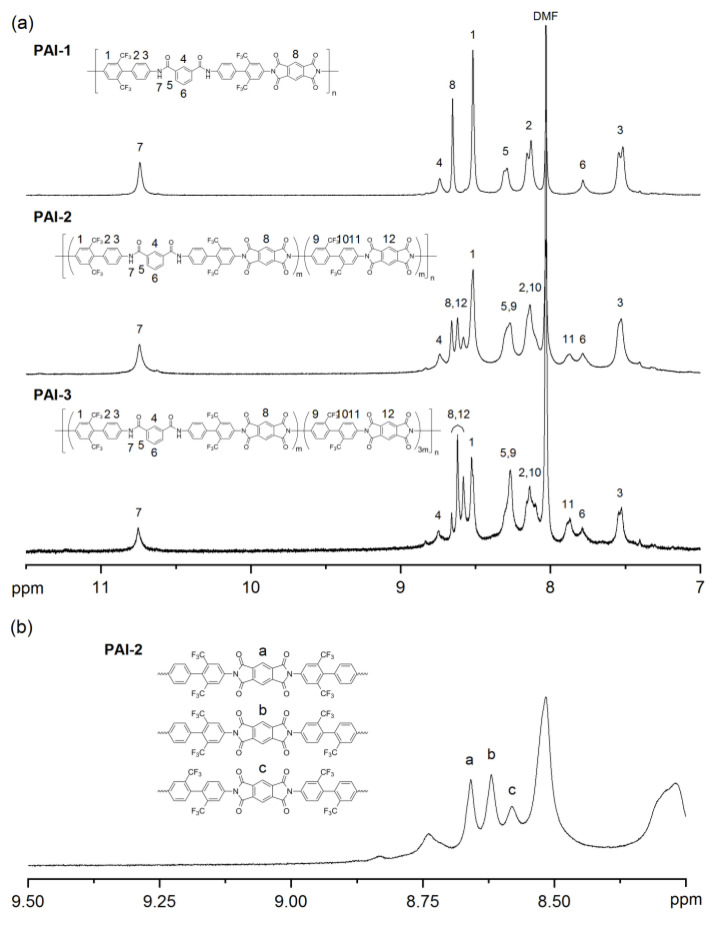
(**a**) ^1^H NMR spectra of the PAIs (DMF-*d*_7_, 25 °C). (**b**) magnified ^1^H NMR of **PAI-2**.

The chemical structures of the PAIs were characterized by ^1^H NMR and FT-IR spectroscopy. ^1^H NMR spectra of the PAIs are presented in Figure 2a. After thermal curing at 300 °C, the proton peaks associated with the unreacted amic acid completely disappeared and all the other proton peaks were well assigned to their predicted structure, which demonstrates the successful preparation of PAIs. In the case of copolymers, the incorporation ratio of two diamine monomer could be determined by comparing the integration value of the corresponding protons, *H*_5_ + *H*_9_ and *H*_3_. The integral ratio of (*H*_5_ + *H*_9_) to *H*_3_ was found to be 1.10:1 for **PAI-2** and 1.95:1 for **PAI-3**. These values were close to the ideal value (1:1 for **PAI-2** and 2:1 for **PAI-3**) and indicate that the copolymers had incorporation ratios close to the monomer feed ratios. Notably, the proton peak from PMDA unit was divided with the different chemical shifts depending on the structures of adjacent diamine unit. ^1^H NMR spectrum of **PAI-2** is magnified in Figure 2b. As illustrated, the resonance peak from a PMDA unit adjacent to **3** appeared further downfield because of the deshielding effect of the electron-withdrawing CF_3_ groups.

**Figure 3:**
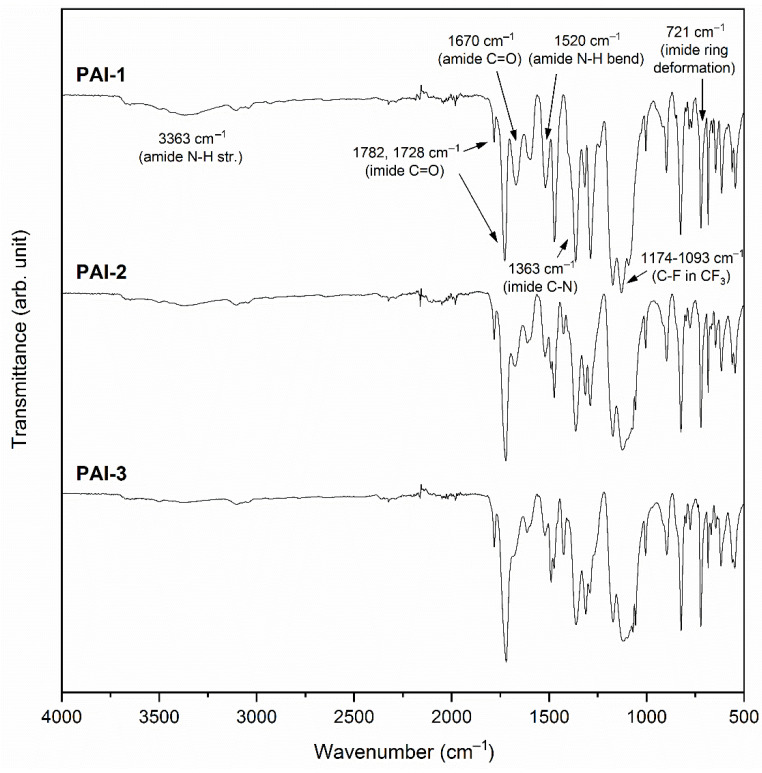
FT-IR spectra of the PAIs.

FT-IR spectra of the PAIs are shown in Figure 3. All PAIs exhibited characteristic absorption bands corresponding to their functional groups around 3363 cm^−1^ (amide N-H stretching), 1782 and 1728 cm^−1^ (imide carbonyl asymmetric and symmetric stretching), 1670 cm^−1^ (amide C=O), 1520 cm^−1^ (amide N-H bending), 1363 cm^−1^ (imide C-N stretching), 1174–1093 cm^−1^ (C-F stretching in CF_3_), and 721 cm^−1^ (imide ring deformation). In addition, from **PAI-1** to **PAI-3** with increasing imide bond content, the relative peak intensity of amide bond to imide bond gradually decreased. This tendency was well matched with the expected polymer structure, and thus further verified the successful formation of PAIs.

### 3.3. Polymer Properties

The solubility of PAIs prepared in this study was tested in various solvents, and the result is summarized in Table 1. Both homopolymer **PAI-1** and copolymer **PAI-2** exhibited good solubility in polar aprotic solvents such as *N*-methyl-2-pyrrolidone, *N*,*N*-dimethylacetamide, and *N*,*N*-dimethylformamide at room temperature in spite of its rigid structures. In addition, **PAI-2** was even soluble in tetrahydrofuran at room temperature. Given the fact that homopolyimides from TFMB/PMDA are insoluble in any organic solvents after thermal curing [24], the good solubility of the PAIs indicates that the incorporation of amide linkages are effective in improving the solubility of polyimides while retaining its rigid character. Meanwhile, **PAI-3** was only soluble in hot organic solvents and quickly became gelated upon cooling. The decreased solubility of **PAI-3** might be attributed to its high chain rigidity and lower amide content.

**Figure 4:**
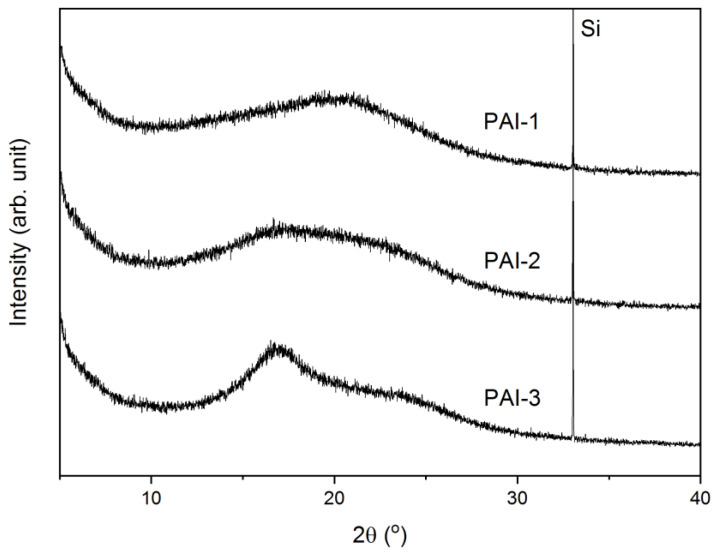
WAXD patterns of the PAIs.

The crystallinity of the PAIs was further investigated by wide-angle X-ray diffraction (WAXD) in order to clarify the reason for the solubility of PAIs, because a crystalline domain can influence polymer solubility [25,26]. As shown in Figure 4, **PAI-1** and **PAI-2** only showed a broad diffraction plateau without a discernible crystalline peak, indicating that those two polymers hold amorphous morphology and thus demonstrate their good organosolubility. On the other hand, **PAI-3** exhibited a certain peak around 17^o^, which indicated that **PAI-3** had a more ordered phase when compared to the other soluble PAIs. Since that certain peak appeared with the incorporation of TFMB and increased with its content from **PAI-1** to **PAI-3**, it could be ascertained that decreased solubility of the **PAI-3** is related to its highly rigid chain structure stems from linear TFMB/PMDA segment, which induced high intermolecular interaction between polymer chains.

**Figure 5:**
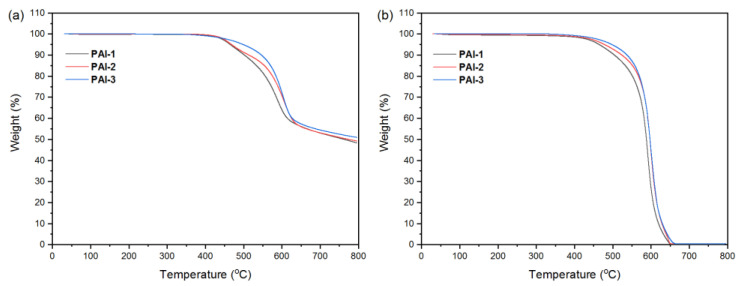
TGA curves of the PAIs in (**a**) nitrogen and (**b**) air at a heating rate of 10 °C/min.

**Figure 6:**
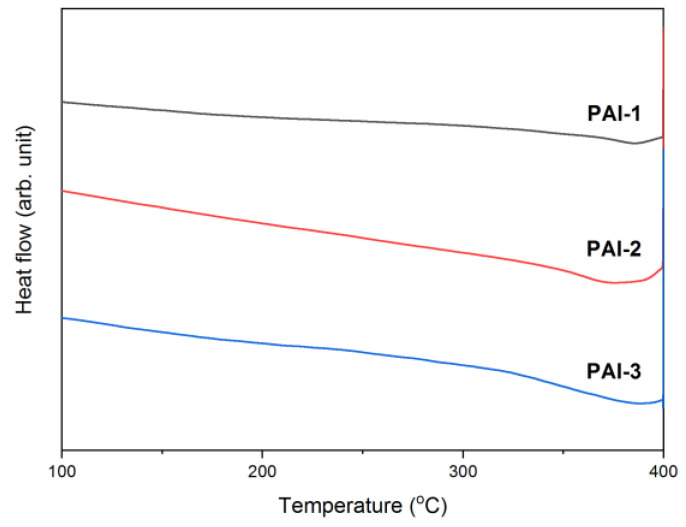
DSC curves of PAIs (second heating scan, heating rate: 10 °C/min, in N_2_).

The thermal properties of the PAIs were evaluated by means of TGA, DSC, and TMA, and the results are summarized in Table 2. TGA results showed that all of the synthesized PAIs possess good thermal stability when the 5% weight loss temperature (*T*_d5_) was in the range of 465–498 °C in N_2_ and 464–497 °C in air, respectively (Figure 5, Figure S9). TGA reveals that PAIs show two weight-loss stages in the range of 400–500 °C and 550–650 °C. It seems that the first weight-loss stage stems from the degradation of main chains through thermolysis of the amide and imide groups, and the second stage is the thermo-oxidative decomposition of aromatic residues in air [27]. Due to the higher thermal stability of imide bonds compared to amide bonds [28], thermooxidative stability of the PAIs was increased with the imide bond content, and as a result, **PAI-3** exhibited the highest *T*_d5_ value close to 500 °C. A survey of all of the PAIs by DSC revealed no endothermic peaks associated with the melting or glass transition in temperatures up to 400 °C (Figure 6, Figure S6–S8). The absence of phase transition of the PAIs stems from their highly rigid structure of which stiff biphenyl units are solely connected by strong amide and imide linkages [29,30].

**Figure 7:**
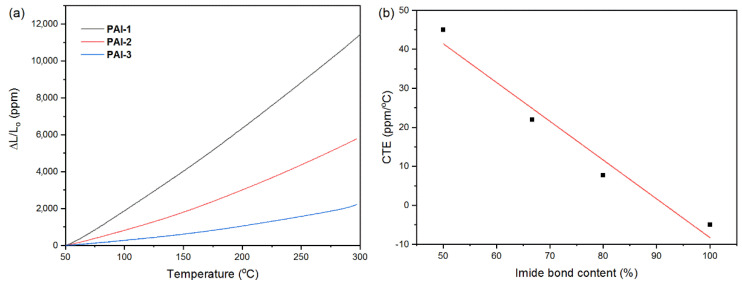
(**a**) TMA curves of the PAIs (3rd heating run, heating rate: 5 °C/min, in N_2_). (**b**) The relation between imide bond content and CTE of the PAIs (CTE data of TFMB/PMDA homopolyimide taken from ref. [24]).

The linear coefficients of thermal expansion (CTEs) of the PAI films were in the range of 7.8 to 44.8 ppm/ °C in the third run. In general, rigid-rod polymers with more rectilinear structures show lower CTE values due to the high parallel chain alignment in the film plane [31]. The PAIs described here also follow this general structure–property relationship, and the CTE of the polymer was sharply decreased with the increasing portion of rigid and linear TFMB/PMDA imide segments (Figure 7a). In addition, it is noteworthy that the CTE value of the PAIs was linearly decreased with the imide bond content (Figure 7b). This linear relationship indicates that thermo-dimensional stability of PAIs could be deliberately controlled via modulating the ratio between diamine monomers within co-PAIs.

Freestanding films of PAIs could be obtained by solution casting from their DMAc or NMP solution (Figure S5). All polymer films exhibited light-brown color due to the presence of a highly electron-deficient PMDA unit which forms a color-inducing interchain charge-transfer (CT) complex between electron-rich diamine units [32]. Nevertheless, the PAI films had enough transparencies to observe the picture placed behind.

Mechanical properties of the PAI films were measured by a UTM (universal testing machine) (Figure S10). PAI-1 showed a tensile modulus of 3.89 MPa and elongation at break of 79.6%, and PAI-2 showed a tensile modulus of 4.27 MPa and elongation at break of 45%.

Optical properties of the PA films were measured by UV-Vis spectrometry and the results are summarized in Table 3. While **PAI-1** and **PAI-2** exhibited moderate transparencies over 80% at 550 nm, **PAI-3** showed relatively less transparency due to its highly rigid and linear chain structure which fortifies the interchain CT interaction. Interestingly, **PAI-2** showed slightly better optical transparency compared to **PAI-1** even though it possesses more linear segments derived from TFMB. It seems that the use of two diamine monomers with different chemical structures caused the lack of uniformity in polymer chain, disrupting their regular arrangement and thus enhancing the optical properties of the polymer film.

The refractive indices and birefringences of the PAI films were investigated by using a prism coupler equipped with laser beams with wavelengths of 633 nm and 1310 nm. Measurement of PAI-3 film was not feasible because it was difficult to obtain a quality film with a flat surface due to the limited solubility of the polymer. As shown in Table 4, the polymers showed low refractive indices (*n*_av_) below 1.6 at both 633 nm and 1310 nm due to the weak molecular polarizability together with low density of the polymer films presumably caused by the abundant CF_3_ groups. The birefringence (Δ*n*) values of the PAIs were calculated as the difference between *n*_TE_ and *n*_TM_ and were in the range of 0.080–0.094 at 633 nm and 0.080–0.094 at 1310 nm. PAI-2 showed a higher birefringence value than PAI-1 because its higher chain rigidity led to high parallel chain alignment in the film plane. The dielectric constant (ε) can be calculated from the refractive index by using Maxwell’s equation, ε ≈ *n*^2^. The ε at 1 MHz was estimated to be ε ≈ 1.10 *n*_av_^2^, including an approximately 10% contribution owing to the infrared absorption [33]. The dielectric constant of the PAI films obtained from the average refractive indices were in the range of 2.79–2.80 at 633 nm and 2.70–2.71 at 1310 nm. The low dielectric constants of PAIs are also attributed to the introduction of low polarizable CF_3_ groups on the main chain.

## 4. Conclusions

New diamide–diamine monomers containing trifluoromethyl groups were synthesized and polymerized with PMDA through solution imidization, followed by thermal curing to produce soluble poly(amide-imide)s. Incorporation of amide linkages together with trifluoromethyl groups into the polyimide backbone effectively enhanced the solubility of the polymers without degrading their thermal properties, and the resulting poly(amide-imide)s exhibited good solubility in polar organic solvents as well as high thermal stabilities. All of the PAIs could be processed into freestanding film by solution casting. In addition, thermal expansion properties of the PAI films could be precisely controlled via modulating the ratio between imide and amide bonds. These solution-processable PAIs with excellent thermal stability are expected to find their application in display and microelectronic devices.

## Figures and Tables

**Table 1:** Solubility of the PAIs.

Solvents	PAI-1	PAI-2	PAI-3
NMP	++	++	+ (gel on r.t.)
DMAc	++	++	+ (gel on r.t.)
DMF	++	++	+ (gel on r.t.)
DMSO	+− (gel on r.t.)	+−	−
*m*-cresol	−	−	−
THF	−	++	−
Anisole	−	−	−
Chloroform	−	−	−
Ethyl acetate	−	−	−
Acetone	−	−	−

Solubility: ++, soluble at room temperature; +, soluble on heating; + −, partially soluble; −, insoluble. Abbreviations: NMP, *N*-methyl-2-pyrrolidone; DMAc, *N*,*N*-Dimethylacetamide; DMF, *N*,*N*-dimethylformamide; DMSO, dimethyl sulfoxide; THF, tetrahydrofuran.

**Table 2:** Thermal properties of the PAIs.

Polymer Code	*T*_d5_ (°C) ^a^		*T*_g_ (°C) ^b^	CTE (ppm/°C) ^c^		Char Yield (%) ^d^
	In N_2_	In Air		2nd Run	3rd Run	
**PAI-1**	465	464	- ^e^	45.0	44.8	48
**PAI-2**	468	478	- ^e^	21.9	21.9	49
**PAI-3**	498	497	- ^e^	7.7	7.8	51

^a^ 5% weight loss temperature, measured by TGA at a heating rate of 10 °C/min. ^b^ measured by DSC (second scan) in N_2_ at a heating rate of 10 °C/min. ^c^ Linear coefficient of thermal expansion, measured by TMA in N_2_ at a heating rate of 5 °C/min. ^d^ Residual weight at 800 °C in nitrogen. ^e^ Not detected up to 400 °C.

**Table 3:** Optical properties of the PAIs.

Polymer Code	Cutoff Wavelength (nm)	Transmittance (%)		Film Thickness (μm)
		at 400 nm	at 550 nm	
**PAI-1**	358	13.0	82.1	~25
**PAI-2**	364	11.8	83.6	~25
**PAI-3**	370	10.6	62.0	~15

**Table 4:** Refractive indices of the PAIs.

λ (nm)	Polymer Code	*n*_TE_ ^a^	*n*_TM_ ^b^	*n*_av_ ^c^	Δ*n* ^d^	ε ^e^	d (μm) ^f^
633	**PAI-1**	1.620	1.540	1.593	0.080	2.79	16.5
	**PAI-2**	1.627	1.533	1.596	0.094	2.80	20.9
	**PAI-3** ^h^	-	-	-	-	-	-
1310	**PAI-1**	1.596	1.516	1.569	0.080	2.71	15.0
	**PAI-2**	1.596	1.512	1.568	0.084	2.70	20.6
	**PAI-3** ^h^	-	-	-	-	-	-

^a^ *n*_TE_: the in-plane refractive index. ^b^ *n*_TM_: the out-of plane refractive index. ^c^ *n*_av_: the average refractive index (*n*_av_ = (2*n*_TE_ + *n*_TM_)/3). ^d^ Δ*n*: birefringence (*n*_TE_ − *n*_TM_). ^e^ Dielectric constant estimated from the refractive index: ε ≈ 1.10*n*_av_^2^. ^f^ Film thickness for the refractive index measured. ^h^ Cannot be measured.

## Supplementary Materials

The following supporting information can be downloaded at: https://www.mdpi.com/article/10.3390/polym14030624/s1, Figure S1: FT-IR spectra of nitro-amine (1), diamide-dinitro (2), diamide-diamine (3) compounds. Figure S2: (a) 1H and (b) 13C NMR spectra of diamide-dinitro compound 2 (DMSO-d6, 25 °C). Figure S3: GPC diagram of PAI-1. Figure S4: GPC diagram of PAI-2. Figure S5: Free standing films of PAI-1 and PAI-2. Figure S6: DSC curves of PAI-1. Figure S7: DSC curves of PAI-2. Figure S8: DSC curves of PAI-3. Figure S9: TGA curves of PAI-1, PAI-2 and PAI-3. Figure S10: Mechanical properties of PAIs. Figure S11: MALDI-TOF of diamide-diamine monomer (3) synthesized in this study.

## References

[1] Malay G., Kash M. (1996). Polyimides: Fundamentals and Applications.

[2] Liaw D.-J., Wang K.-L., Huang Y.-C., Lee K.-R., Lai J.-Y., Ha C.-S. (2012). Advanced polyimide materials: Syntheses, physical properties and applications. Prog. Polym. Sci..

[3] Chung I.S., Kim S.Y. (2000). Soluble Polyimides from Unsymmetrical Diamine with Trifluoromethyl Pendent Group. Macromolecules.

[4] Yang C.-P., Hsiao S.-H., Yang H.-W. (2000). Organosoluble optically transparent poly(ether imide)s based on atert-butylhydroquinone bis(ether anhydride). Macromol. Chem. Phys..

[5] Choi H., Chung I.S., Hong K., Park C.E., Kim S.Y. (2008). Soluble polyimides from unsymmetrical diamine containing benzimidazole ring and trifluoromethyl pendent group. Polymer.

[6] Kim S.D., Kim S.Y., Chung I.S. (2013). Soluble and transparent polyimides from unsymmetrical diamine containing two trifluoromethyl groups. J. Polym. Sci. Part A Polym. Chem..

[7] Yi L., Huang W., Yan D. (2017). Polyimides with side groups: Synthesis and effects of side groups on their properties. J. Polym. Sci. Part A Polym. Chem..

[8] O’Harra K., Kammakakam I., Devriese E., Noll D., Bara J., Jackson E. (2019). Synthesis and Performance of 6FDA-Based Polyimide-Ionenes and Composites with Ionic Liquids as Gas Separation Membranes. Membranes.

[9] Dodda J.M., Bělský P. (2016). Progress in designing poly(amide imide)s (PAI) in terms of chemical structure, preparation methods and processability. Eur. Polym. J..

[10] Liaw D.-J., Liaw B.-Y. (1998). Synthesis and properties of new polyamides derived from 1,4-bis(4-aminophenoxy)-2,5-di-tert-butylbenzene and aromatic dicarboxylic acids. J. Polym. Sci. Part A Polym. Chem..

[11] Do Lee Y., Kim K., OK Y., Kim M., Chang J.-H. (2016). Syntheses and Characterizations of Colorless and Transparent Poly(amide imide) Copolymer. Polym. Korea.

[12] Yin J., Mao D., Fan B. (2021). Copolyamide-Imide Membrane with Low CTE and CME for Potential Space Optical Applications. Polymers.

[13] Hasegawa M., Ishigami T., Ishii J., Sugiura K., Fujii M. (2013). Solution-processable transparent polyimides with low coefficients of thermal expansion and self-orientation behavior induced by solution casting. Eur. Polym. J..

[14] Kim S.D., Lee B., Byun T., Chung I.S., Park J., Shin I., Ahn N.Y., Seo M., Lee Y., Kim Y. (2018). Poly(amide-imide) materials for transparent and flexible displays. Sci. Adv..

[15] Murad A.R., Iraqi A., Aziz S.B., Hi H., Abdullah S.N., Brza M.A., Abdulwahid R.T. (2020). Influence of Fluorine Substitution on the Optical, Thermal, Electrochemical and Structural Properties of Carbazole-Benzothiadiazole Dicarboxylic Imide Alternate Copolymers. Polymers.

[16] James M. (1985). Engineering Thermoplastics: Properties and Applications.

[17] Chris D., Roger R., Myer K. (2011). Applied Plastics Engineering Handbook.

[18] Patil P.S., Pal R.R., Salunkhe M.M., Maldar N.N., Wadgaonkar P.P. (2007). Synthesis of aromatic poly(amide-imide)s from novel diimide-diacid (DIDA) containing sulphone and bulky pendant groups by direct polycondensation with various diamines. Eur. Polym. J..

[19] Bai L., Zhai L., He M., Wang C., Mo S., Fan L. (2019). Preparation of heat-resistant poly(amide-imide) films with ultralow coefficients of thermal expansion for optoelectronic application. React. Funct. Polym..

[20] Javadi A., Shockravi A., Rafieimanesh A., Malek A., Ando S. (2015). Synthesis and structure-property relationships of novel thiazole-containing poly(amide imide)s with high refractive indices and low birefringences. Polym. Int..

[21] Li W., Qian X., Shi H., Zhou W., Cai Y., Liu Y., Shen K. (2017). Synthesis and properties of novel soluble poly(amide-imide)s with different pendant substituents. J. Polym. Sci. Part A Polym. Chem..

[22] Özarslan Ö., Yilmaz T., Yildiz E., Fiedeldei U., Kuyulu A., Güngör A. (1997). The preparation of perfectly alternating poly(amide-imide)s via amide unit containing new diamine. J. Polym. Sci. Part A Polym. Chem..

[23] Hasegawa M., Watanabe Y., Tsukuda S., Ishii J. (2016). Solution-processable colorless polyimides with ultralow coefficients of thermal expansion for optoelectronic applications. Polym. Int..

[24] Matsuura T., Hasuda Y., Nishi S., Yamada N. (1991). Polyimide derived from 2,2′-bis(trifluoromethyl)-4,4′-diaminobiphenyl. 1. Synthesis and characterization of polyimides prepared with 2,2′-bis(3,4-dicarboxyphenyl)hexafluoropropane dianhydride or pyromellitic dianhydride. Macromolecules.

[25] Kim S.D., Ka D., Chung I.S., Kim S.Y. (2012). Poly(arylene ether)s with Low Refractive Indices: Poly(biphenylene oxide)s with Trifluoromethyl Pendant Groups via a Meta-Activated Nitro Displacement Reaction. Macromolecules.

[26] Lee S., Jeong R., Seo M., Lee H.-S. (2020). Double-activated nucleophilic aromatic substitution polymerization by bis-ortho-trifluoromethyl groups to soluble para-poly(biphenylene oxide). Polymer.

[27] Zhang K., Ishida H. (2015). Smart Synthesis of High-Performance Thermosets Based on ortho-Amideâ-Imide Functional Benzoxazines. Front. Mater..

[28] Pramoda K.P., Chung T.S., Liu S.L., Oikawa H., Yamaguchi A. (2000). Characterization and thermal degradation of polyimide and polyamide liquid crystalline polymers. Polym. Degrad. Stab..

[29] Hong W., Yuan L., Ma Y., Cui C., Zhang H., Yang S., Sun W.-H. (2021). Resin Transfer Moldable Fluorinated Phenylethynyl-Terminated Imide Oligomers with High Tg: Structure-Melt Stability Relationship. Polymers.

[30] Hasegawa M., Hishiki T. (2020). Poly(ester imide)s Possessing Low Coefficients of Thermal Expansion and Low Water Absorption (V). Effects of Ester-linked Diamines with Different Lengths and Substituents. Polymers.

[31] Ishige R., Masuda T., Kozaki Y., Fujiwara E., Okada T., Ando S. (2017). Precise Analysis of Thermal Volume Expansion of Crystal Lattice for Fully Aromatic Crystalline Polyimides by X-ray Diffraction Method: Relationship between Molecular Structure and Linear/Volumetric Thermal Expansion. Macromolecules.

[32] Hasegawa M. (2017). Development of Solution-Processable, Optically Transparent Polyimides with Ultra-Low Linear Coefficients of Thermal Expansion. Polymers.

[33] Wooten F. (1972). Optical Properties of Solids.

