# Supplementary material for: Soluble Poly(amide-imide)s from Diamide–Diamine Monomer with Trifluoromethyl Groups
Source: Polymers (Basel). 2022 Feb 6;14(3):624. doi: 10.3390/polym14030624 (PMC8840246; doi:10.3390/polym14030624)
Supplement: Supplementary file 1 [file polymers-14-00624-s001.zip › polymers-1566930-supplementary.pdf]

Soluble Poly(amide-imide)s from Diamide-Diamine with Trifluoromethyl Groups

Taejoon Byun, Seong Jong Kim and Sang Youl Kim*

Department of Chemistry, Korea Advanced Institute of Science and Technology (KAIST), Daejeon 34141, Korea.
* Correspondence: kimsy@kaist.ac.kr; Tel.: (+)82-42-350-2834.

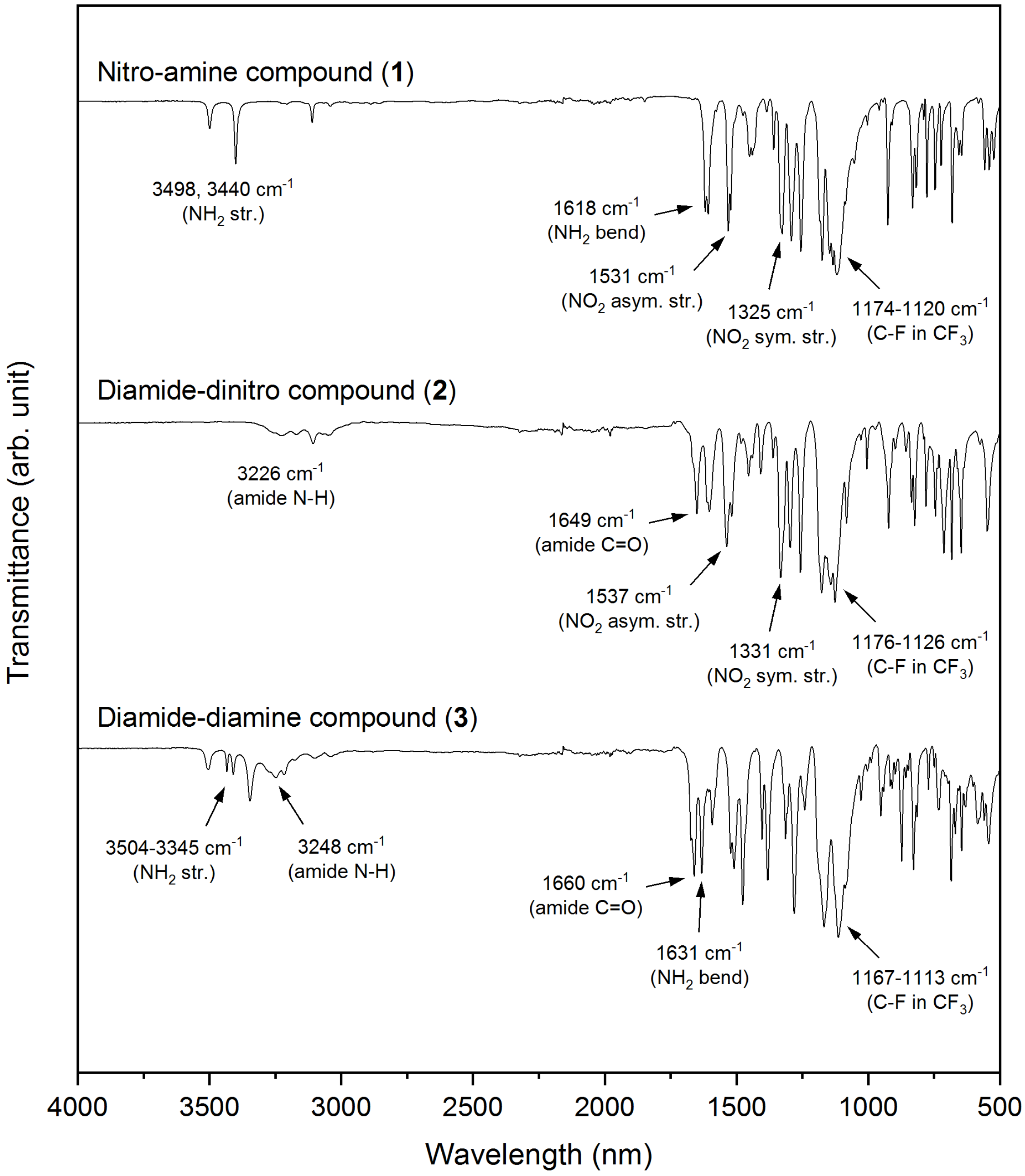

Figure S1. FT-IR spectra of nitro-amine (**1**), diamide-dinitro (**2**), diamide-diamine (**3**) compounds.

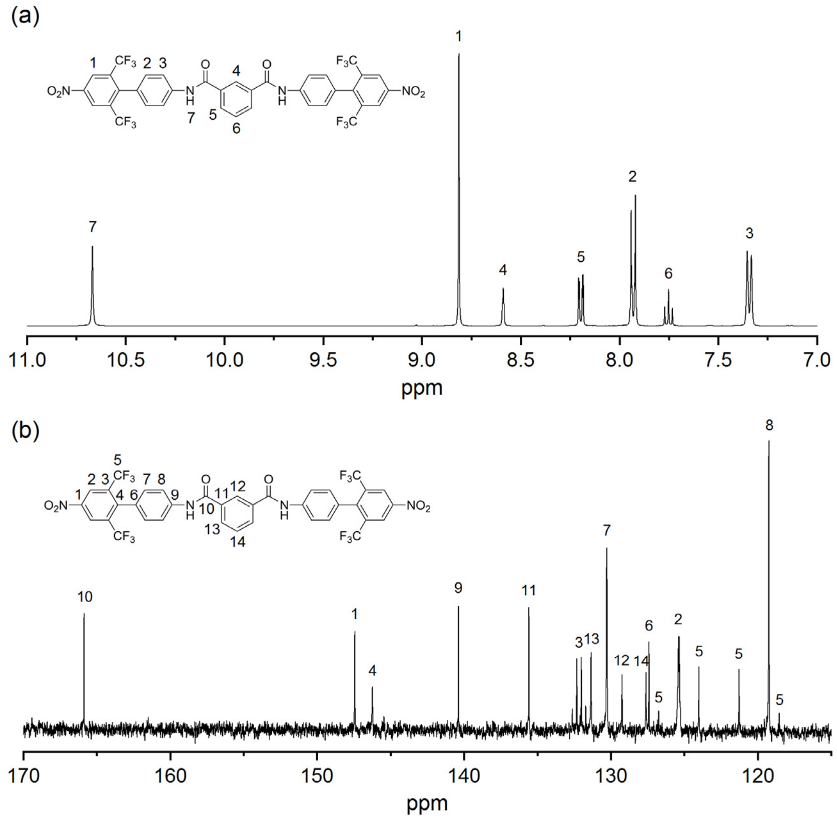

Figure S2. (a) ^{1}H and (b) ^{13}C NMR spectra of diamide-dinitro compound **2** (DMSO-d_6, 25°C).

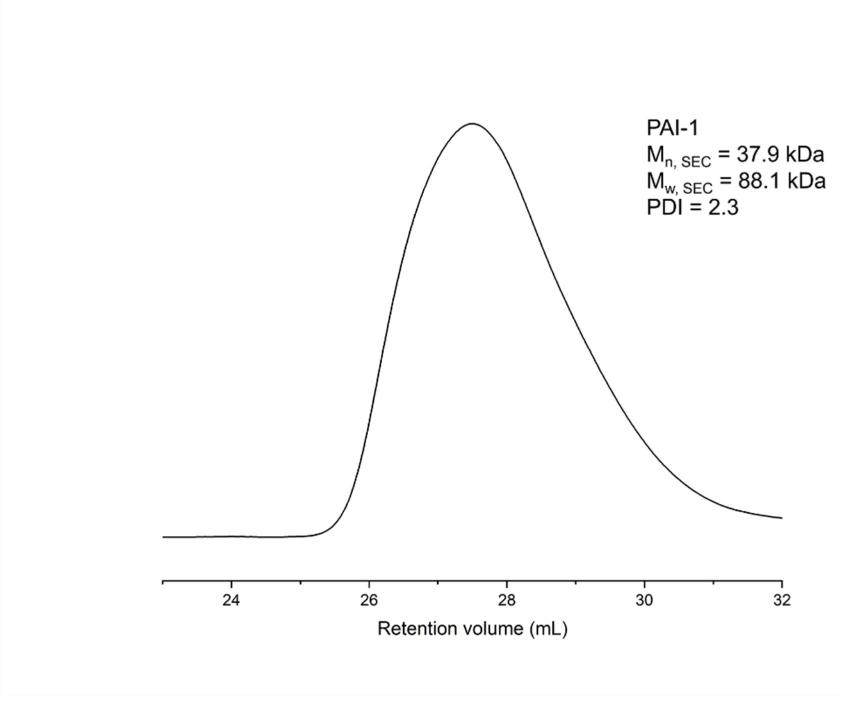

Figure S3. GPC diagram of **PAI-1**.

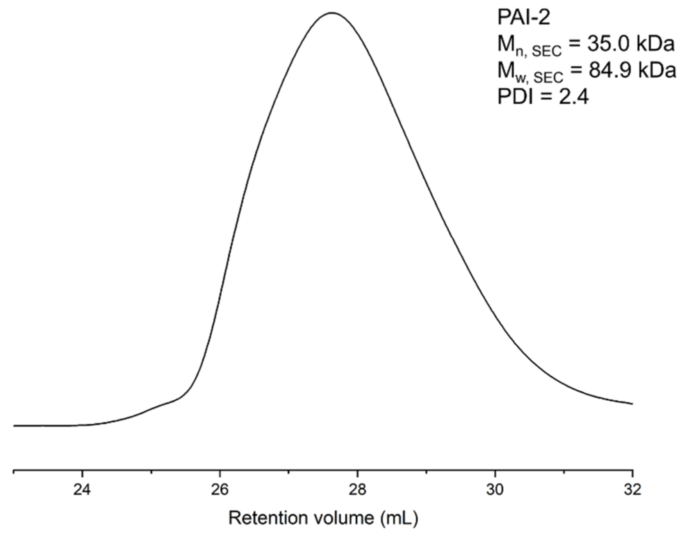

Figure S4. GPC diagram of **PAI-2**.

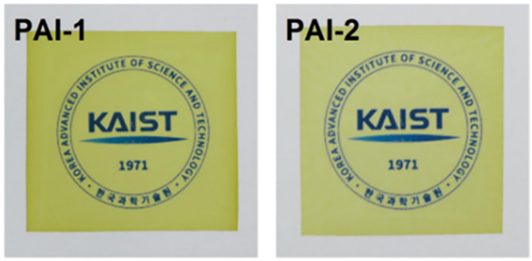

Figure S5. Free standing films of **PAI-1** and **PAI-2**.

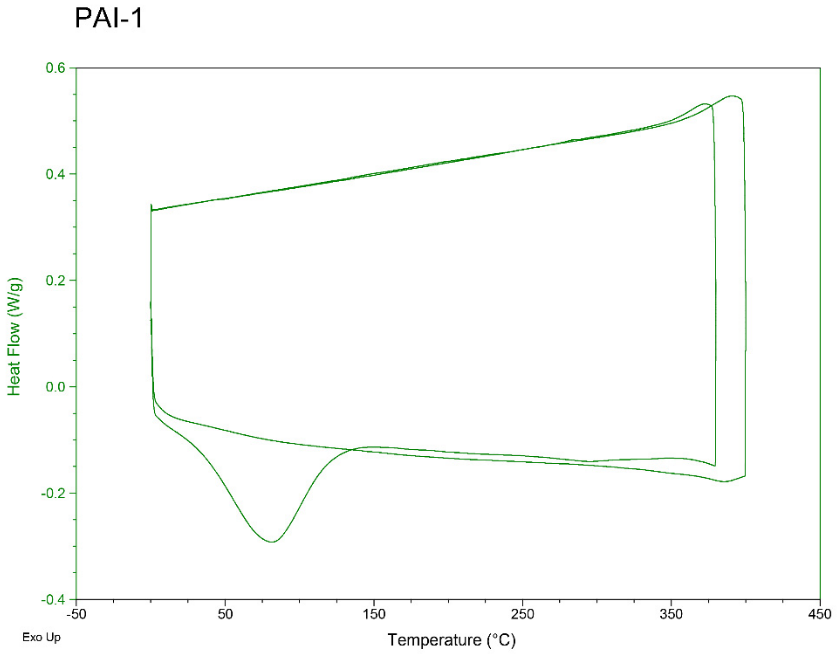

Figure S6. DSC curves of **PAI-1**.

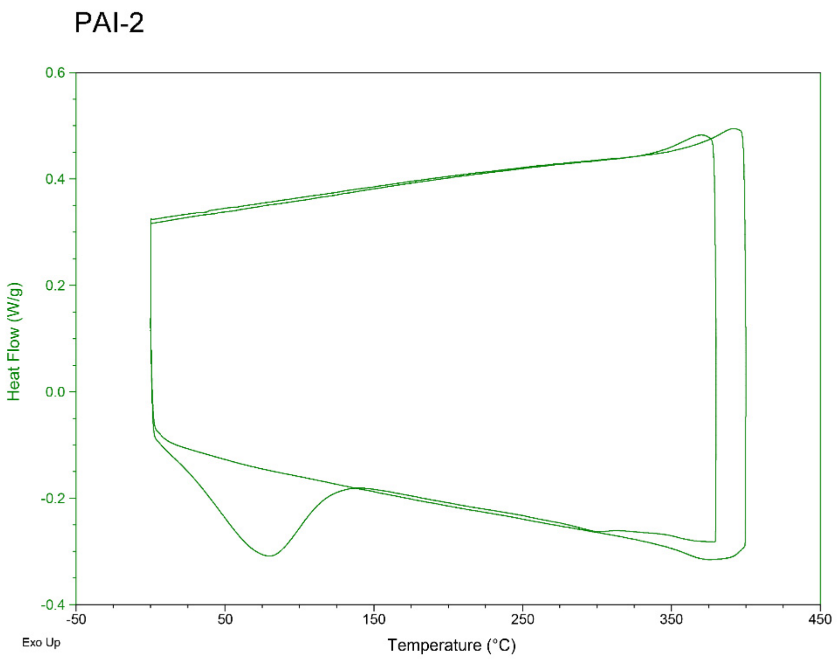

Figure S7. DSC curves of **PAI-2**.

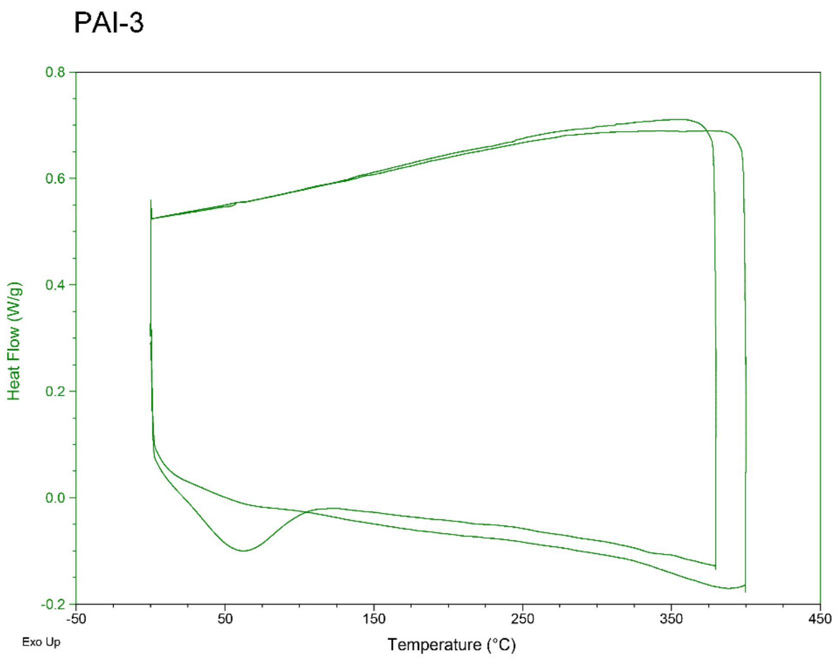

Figure S8. DSC curves of **PAI-3**.

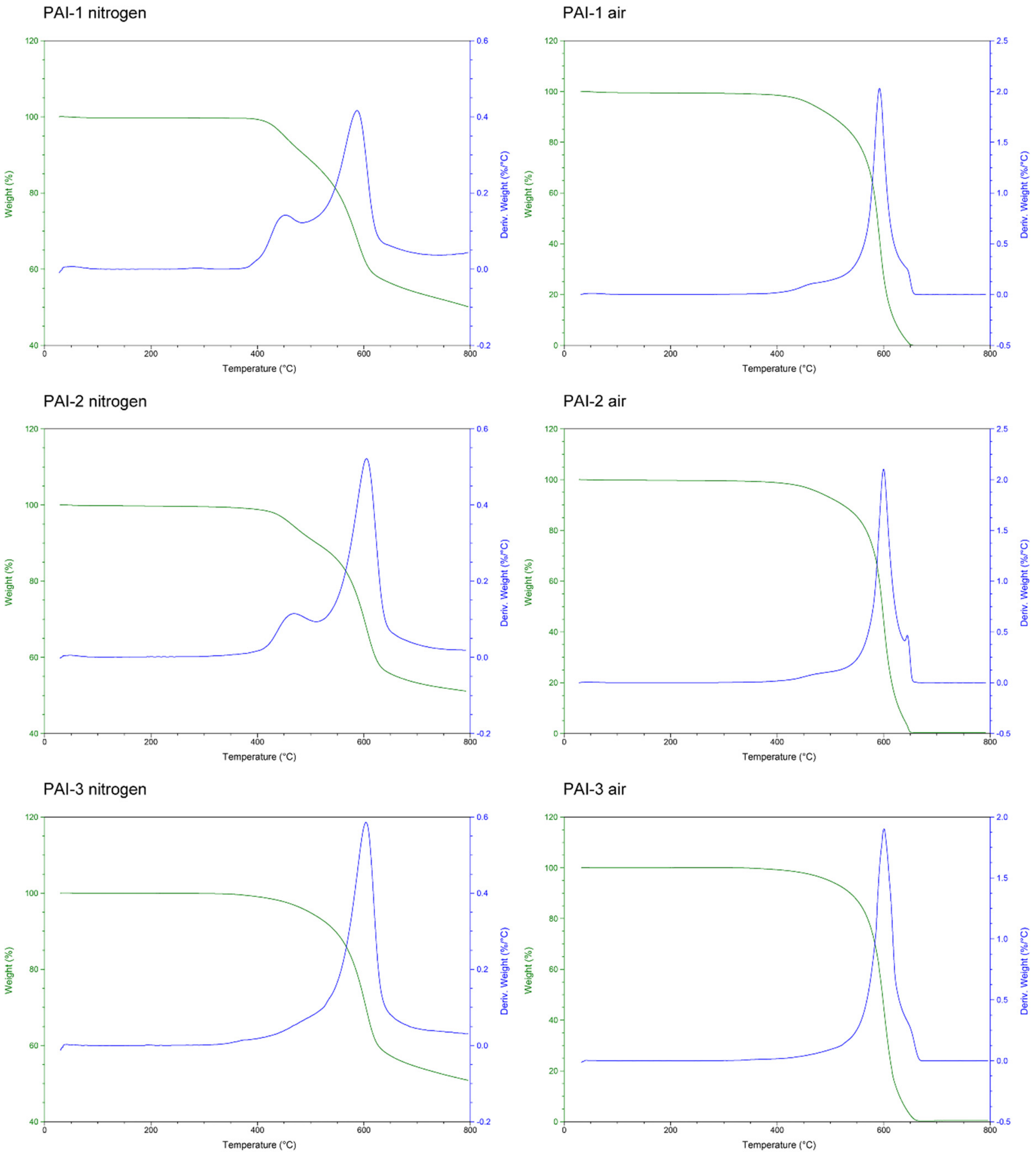

Figure S9. TGA curves of **PAI-1**, **PAI-2** and **PAI-3**.

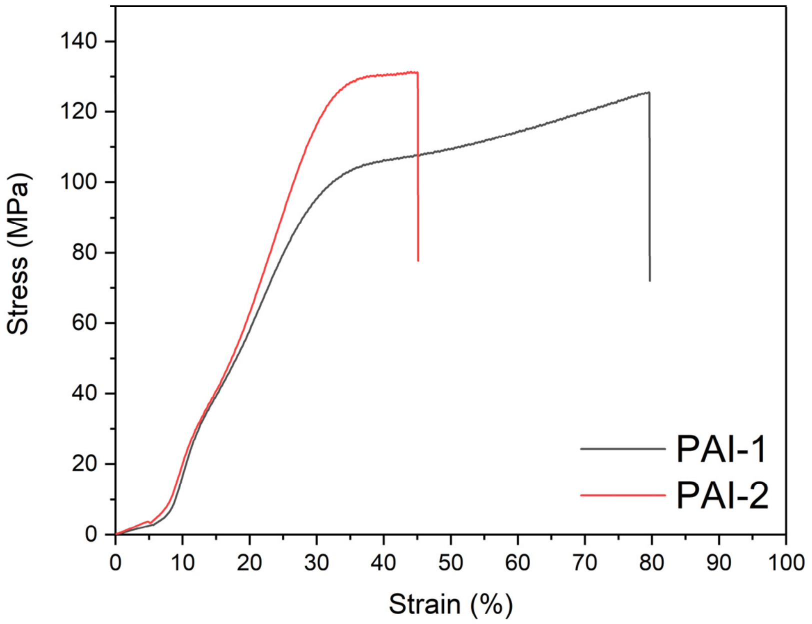

Figure S10. Mechanical properties of PAIs.

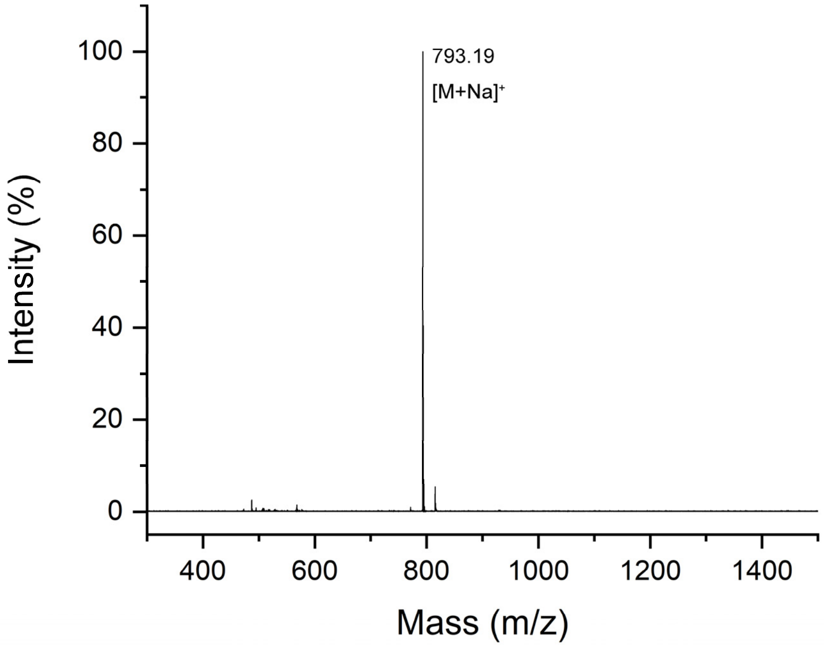

Figure S11. MALDI-TOF of diamide-diamine monomer synthesized in this study (**3**).
